# Remnant Cholecystitis After Subtotal Cholecystectomy: A Case Report

**DOI:** 10.7759/cureus.71719

**Published:** 2024-10-17

**Authors:** Heather L Mateja, Danielle A Rowe, Allen Tsai, Pablo Giuseppucci

**Affiliations:** 1 General Surgery, American University of Antigua, Osbourn, ATG; 2 General Surgery, Western Reserve Health Education/Northeast Ohio Medical University (NEOMED), Warren, USA

**Keywords:** acute cholecystitis, family practice, recurrent cholecystitis, remnant cholecystectomy, remnant cholecystitis, right upper quadrant abdominal pain, subtotal cholecystectomy, ultrasound

## Abstract

Remnant cholecystitis is a rare complication following subtotal cholecystectomy (STC), particularly when the reconstituting technique is used, which leaves a portion of the gallbladder behind. This remnant can become inflamed due to recurrent or retained gallstones. We present the case of a 39-year-old female who required a completion cholecystectomy 11 years after her initial STC due to severe recurrent right upper quadrant (RUQ) pain, nausea, and vomiting with an ultrasound that revealed cholelithiasis. This case highlights the need for increased awareness of remnant cholecystitis, better diagnostic approaches, and standardized management guidelines to prevent long-term complications. Further research is necessary to improve the treatment of this rare condition and optimize patient outcomes.

## Introduction

A subtotal cholecystectomy (STC) is a surgical procedure that involves partial resection of the gallbladder [1,2]. This bailout technique is employed most commonly when surgeons encounter difficulties accessing Calot’s triangle due to dense adhesions [2,3]. STCs are classified into two types - fenestrating and reconstituting - based on whether the remaining portion of the gallbladder is left open or closed [3,4]. In the reconstituting subtype, the lower end of the gallbladder is closed off. This reduces the incidence of postoperative fistulas but creates a remnant gallbladder, which may result in the recurrence of symptomatic cholecystolithiasis [3,4]. In the fenestrating subtype, the gallbladder is left unobstructed, but the cystic duct may be sutured internally [3]. This subtype has a higher incidence of postoperative biliary fistula but does not appear to be associated with recurrent cholecystolithiasis [3,4].

Remnant cholecystitis is a rare complication of subtotal cholecystectomy, which is most seen in the reconstituting technique due to the creation of a gallbladder stump [1-4]. This rare complication was noted in 4% of patients in a 2023 review by Al-Azzawi et al. with 2% requiring total cholecystectomy [1]. This emphasizes the need for further investigation into this complication. Given the limited research on this condition and the absence of standardized treatment guidelines, we present the case of a 39-year-old female patient with a past medical history (PMH) of STC, who was referred by her primary care physician (PCP) for severe and recurrent right upper quadrant pain (RUQ) nausea and vomiting requiring a completion cholecystectomy 11 years after the index operation. We aim to raise awareness of this rare condition and highlight the need for clearer clinical guidelines and further research to optimize its management and improve patient outcomes.

## Case presentation

**Figure 1:**
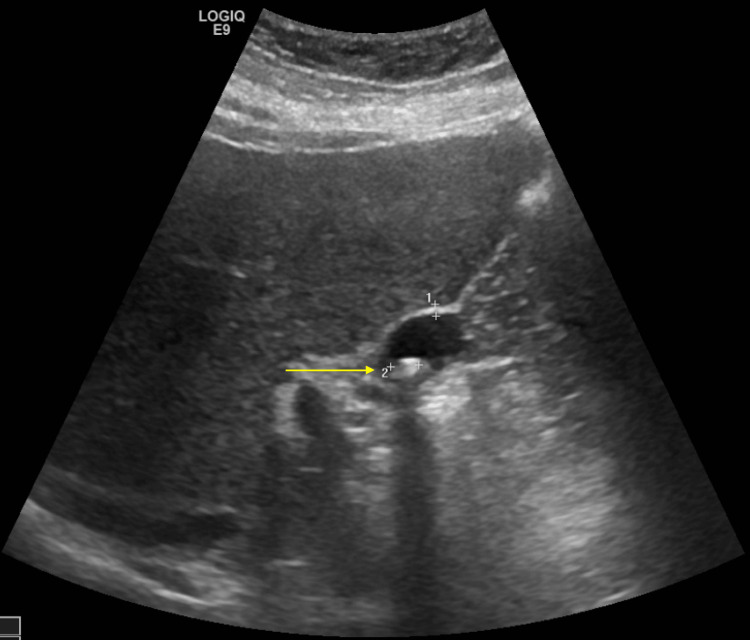
Right upper quadrant ultrasound showing a gallbladder remnant with wall thickness of 1 mm and the presence of an approximately 2 mm gallstone (yellow arrow). Multiple shadowing stones are noted. No pericholecystic fluid is seen. Sonographic Murphy's sign is negative.

A 39-year-old female presented to our hospital for a completion cholecystectomy due to recurrent right upper quadrant (RUQ) pain, nausea, and vomiting, following a history of a prior cholecystectomy performed in 2013. Since her initial surgery, the patient reported never feeling fully recovered, with persistent RUQ pain radiating to her back. She was referred to our surgical team by her primary care physician (PCP) after an ultrasound revealed cholelithiasis, despite her previous cholecystectomy (Figure 1). Her past medical history is otherwise remarkable for prediabetes, with a last hemoglobin A1c of 5.9%, obesity with a BMI of 37.6 kg/m^2^, and hypertension without any other noted surgical procedures. All recent laboratory values had been within normal limits.

The patient described episodes of nausea and vomiting severe enough to necessitate stopping her car to vomit. Her pain was intermittent, beginning in her stomach and RUQ, then radiating to her back, with no specific aggravating factors. She denied associated symptoms such as fever, chills, constipation, or diarrhea. Her prior subtotal cholecystectomy had been initiated laparoscopically but converted to open due to a difficult gallbladder. She recalled emerging from surgery with a right upper quadrant drain that drained bile-like fluid. Notably, the index surgery took place two weeks postpartum.

Based on her symptoms and positive imaging, it was decided to proceed with robotic-assisted laparoscopic removal of the gallbladder remnant. Extensive adhesiolysis was required for over 90 minutes to access the gallbladder fossa, where the remnant gallbladder was identified. The cystic duct was traced to its entry into the common bile duct. Notably, there were no clips placed on the cystic duct, but there were prior surgical clips embedded within the gallbladder wall. An indocyanine green cholangiogram facilitated safe dissection and accurate recognition of biliary structures. The dissection was complicated by abundant scar tissue from the prior operation. Despite the challenging anatomy, the critical view of safety was achieved via a top-down approach, and the cystic duct and artery were triple-clipped and divided.

**Figure 2:**
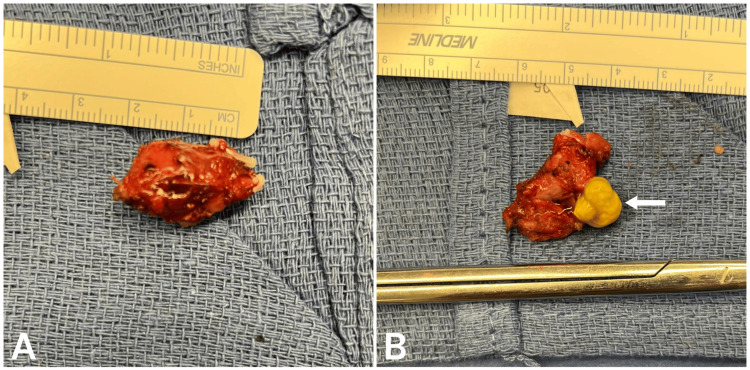
Surgical specimen (A) gallbladder remnant measuring 0.9 cm in length and 1 cm in diameter, and (B) opened gallbladder remnant with large gallstone (white arrow) measuring 0.9 x 0.8 x 0.8 cm

The gallbladder remnant was removed, placed in an Endocatch bag, and retrieved without incident. The patient was awakened from anesthesia and transferred to recovery in stable condition. The specimen (Figure 2) consisted of a pink, tubular remnant of gallbladder tissue measuring 0.9 cm in length and 1 cm in diameter, with a surgical clip at one pole. An incision revealed a light pink, smooth lumen. Additionally, a pale yellow, irregular gallstone measuring 0.9 x 0.8 x 0.8 cm was retrieved. The patient recovered from surgery without incident and was discharged the same day. The patient followed up in the office two weeks later, reporting complete resolution of symptoms and no complications from surgery. Her PCP reports the patient has maintained good health and denies any recurrence of symptoms six months postoperatively.

## Discussion

STCs are generally performed in cases with severe cholecystitis, adhesions in the cholecystohepatic triangle, and impacted stone in Hartman’s pouch [1,5]. The technique is employed in cases where access to critical anatomical structures is compromised, thereby minimizing the risk of bile duct injury [1-3]. In the short term, this approach is a safer alternative to total cholecystectomy [1,2]. However, STC is associated with long-term complications, including remnant cholecystitis [1,2,5]. Specifically, remnant cholecystitis is most seen in the reconstituting technique [3,4]. In this technique, the lower part of the gallbladder is left attached to the liver bed and closed off with staples or sutures, creating a residual space that facilitates future stone formation [3,5].

Remnant cholecystitis is thought to be caused by severe adhesions and fibrosis, and recurrent or retained stones in the remnant gallbladder [5-7]. This was observed in our patient, who presented with cholelithiasis observed both on ultrasound and intraoperatively. Extensive adhesions and fibrosis were also noted, reinforcing the etiological role of these factors. Recent studies have suggested that there is a direct correlation between the diameter of the stump created post-surgery and long-term complications [5-7]. Larger gallbladder stumps were found to be associated with more long-term complications [5-7]. Despite this correlation, there are currently no formal guidelines dictating the optimal size of the remnant gallbladder following STC. The current recommendation is to remove as much of the gallbladder wall as possible and ensure the complete removal of any stones [4]. However, in severe cases with dense adhesions and fibrosis, this may be a difficult task for the surgeon, to avoid a common bile duct (CBD) injury, a larger remnant may be left behind, thus creating the opportunity for future complications [1]. For direct measurement of the remnant gallbladder, a magnetic resonance cholangiopancreatography (MRCP) is required [5]. Our patient did not have one completed prior to the operation, therefore we cannot elucidate on the exact dimensions of the remnant gallbladder.

Patients with remnant cholecystitis typically present with symptoms similar to acute cholecystitis. These include abdominal pain, most commonly localized to the right upper quadrant and epigastric regions, as was seen in our patient [5,7,8]. Other symptoms, such as dyspepsia, nausea, vomiting, chills, and back pain, have also been reported [5,8].

Diagnostic modalities include right upper quadrant ultrasound and computed tomography (CT) scan, both of which can reveal a dilated remnant gallbladder [5,8,9]. Ultrasound is recommended, as it is inexpensive, the least invasive, and has a reported sensitivity of 81% for remnant gallbladders [9]. To visualize retained stones within the remnant gallbladder, the most accurate techniques are magnetic resonance imaging (MRI) and MRCP. These methods offer detailed insights into the anatomy of the biliary system and any related abnormalities [5,9].

Definitive treatment of remnant cholecystitis is the removal of the remnant [9]. In the acute setting, surgery may be difficult due to the adhesions and fibrosis from past cholecystitis and surgery [5,8]. Bridging to surgery may be required in some cases; this can be achieved via percutaneous transhepatic gallbladder drainage or endoscopic gallbladder drainage [5]. Both procedures are incredibly difficult operations; the size of the remnant also makes accessing difficult [5,8].

This case highlights the significant gaps in available data and the absence of clear guidelines for managing remnant cholecystitis. We present this case to underscore the need for further research and the development of standardized guidelines for the management of this rare but challenging complication.

## Conclusions

This case highlights the challenges and complexities associated with remnant cholecystitis, a rare but significant complication of subtotal cholecystectomy, particularly in patients who undergo the reconstituting technique. Our patient’s recurrent right upper quadrant pain, accompanied by ultrasound-confirmed cholelithiasis and extensive intraoperative adhesions, exemplifies the difficulty in managing this condition. Although subtotal cholecystectomy is often employed to reduce the risk of bile duct injury in cases with severe inflammation or dense adhesions, it is not without long-term complications. Definitive treatment involves removing the remnant gallbladder, although this can be technically demanding due to postoperative fibrosis. This case underscores the need for further research into optimal management strategies and the development of guidelines to minimize the incidence of remnant cholecystitis and its associated complications. By increasing awareness and improving diagnostic and surgical approaches, future complications could be better anticipated and managed.
